# Chiasmatic and achiasmatic inverted meiosis of plants with holocentric chromosomes

**DOI:** 10.1038/ncomms6070

**Published:** 2014-10-08

**Authors:** Gabriela Cabral, André Marques, Veit Schubert, Andrea Pedrosa-Harand, Peter Schlögelhofer

**Affiliations:** 1 Department of Botany, Laboratory of Plant Cytogenetics and Evolution, Federal University of Pernambuco, Rua Nelson Chaves s/n, Recife, Pernambuco 50670-420, Brazil; 2 Department of Chromosome Biology, Max F. Perutz Laboratories, University of Vienna, Dr Bohr-Gasse 9, Vienna A-1030, Austria; 3 Leibniz Institute of Plant Genetics and Crop Plant Research, Corrensstraße 3, Gatersleben 06466, Germany

## Abstract

Meiosis is a specialized cell division in sexually reproducing organisms before gamete formation. Following DNA replication, the canonical sequence in species with monocentric chromosomes is characterized by reductional segregation of homologous chromosomes during the first and equational segregation of sister chromatids during the second meiotic division. Species with holocentric chromosomes employ specific adaptations to ensure regular disjunction during meiosis. Here we present the analysis of two closely related plant species with holocentric chromosomes that display an inversion of the canonical meiotic sequence, with the equational division preceding the reductional. In-depth analysis of the meiotic divisions of *Rhynchospora pubera* and *R. tenuis* reveals that during meiosis I sister chromatids are bi-oriented, display amphitelic attachment to the spindle and are subsequently separated. During prophase II, chromatids are connected by thin chromatin threads that appear instrumental for the regular disjunction of homologous non-sister chromatids in meiosis II.

## Abstract (web summary)

The absence of a defined centromere in organisms with holocentric chromosomes presents particular problems for the control of chromosome segregation during meiosis. Cabral *et al.* present evidence that two plant species overcome this challenge by inverting the conventional sequence of meiotic divisions.

## Graphical abstract

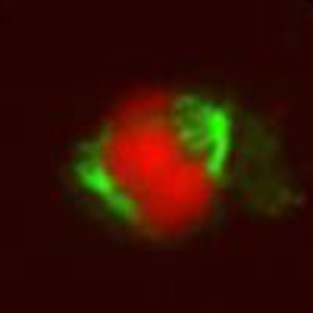

Meiosis is a special type of cell division that occurs in sexually reproducing organisms and results in the formation of gametes. DNA replication is followed by two rounds of chromosome segregation, thereby halving the genomic content. The canonical sequence of events is characterized by sister centromeres segregating together during the first (reductional) division and then separating during the second meiotic (equational) division. Regular chromosome disjunction during meiosis I depends on physical connections between homologous non-sister chromatids. These connections, termed chiasmata, correspond to regions that have undergone interhomologue recombination. Recombined DNA strands together with sister chromatid cohesion hold the homologous chromosomes together until the transition from metaphase I to anaphase I. At anaphase I onset, cohesion of chromosome arms is released but maintained in proximity of sister centromeres. The sister centromeres are co-oriented and attached to the same spindle. In contrast, in metaphase II, sister centromeres are oriented in a bipolar manner and are attached to different spindles resulting in sister centromere separation^1^,^2^.

Meiotic recombination is initiated by the formation of DNA double-strand breaks (DSBs) catalysed by the conserved Spo11 protein (together with additional factors)^3^,^4^. The break ends are subsequently processed into single-stranded DNA overhangs, which invade an intact DNA duplex of the homologous chromosome. This repair process is mediated by RecA-like proteins (Rad51 and Dmc1). Only some of these interhomologue interactions lead to mutual exchange of chromosome parts, thereby re-shuffling genetic information as well as generating physical links (chiasmata)^5^,^6^. The interhomologue interactions support pairing of homologous chromosomes. Stabilization of chromosome pairing is achieved during synapsis, a process in which the axes of homologous chromosomes, comprising meiosis-specific proteins such as Hop1 (named ASY1 in higher plants), are linked together by proteins of the central element such as Zip1 (known as ZYP1 in higher plants) giving rise to a proteinaceous structure, named synaptonemal complex (SC)^7^.

Several studies provided evidence that there is an interdependent relationship between the process of DSB formation, meiotic recombination, chromosome pairing and synapsis in most organisms^8^,^9^. In this sense, DSB formation and subsequent repair have been found to be important for both pairing and synapsis in yeast, mouse and plants such as maize and *Arabidopsis*. In contrast, pairing and synapsis in the worm *Caenorhabidits elegans* and the fly *Drosophila melanogaster* are independent of DSB formation^10^,^11^. In *C. elegans* these processes rely on specific chromosomal regions that associate with chromosome-specific zinc-finger proteins^12^,^13^. Interestingly, in *Drosophila* males, chromosomes segregate reductionally even without the formation of chiasmata (and also chromosome 4 in *Drosophila* females), which has been explained by interactions of heterochromatic regions, at least in the case of sex chromosomes and the autosome 4 (refs 14, 15, 16).

The reductional segregation of homologous chromosomes in anaphase I also relies on monopolar attachment of sister kinetochores to the spindle. Together with cohesion maintenance at the centromeric region, monopolar attachment of sister kinetochores promotes the joint migration of sister chromatids to the same pole at anaphase I^17^,^18^,^19^. In plants and budding yeast, monopolar attachment is promoted by specific kinetochore proteins that bridge the two sister kinetochores during meiosis I (refs 20, 21). Cohesion loss along the chromosome arms but not at the centromeric region allows homologues to segregate at anaphase I, while preserving sister chromatid–centromere association. Protection of cohesion at the centromeric region during meiosis I depends on a specific protein that localizes to centromeres and prevents the cleavage of a cohesin subunit and therefore ensures that sister chromatids remain attached to each other until anaphase II (ref. 22).

On the basis of the extension of the kinetochore region, chromosomes are classified into two major types: monocentric chromosomes with a clearly localized and restricted kinetochore region, and holocentric chromosomes with a more diffused kinetochore that spans the length of condensed chromosomes. Holocentric chromosomes are present in a number of taxa, including nematode worms, such as *Parascaris* and *Caenorhabditis,* several insects orders, such as Odonata and Heteroptera, and higher plants, such as the Cyperaceae and Juncaceae families^23^,^24^,^25^. While neutral for mitotic divisions, the presence of a holocentric kinetochore could impose obstacles to the particular dynamics of cohesion loss in meiosis I that releases chromosome arms but keeps sister centromeres together^26^,^27^. In *C. elegans* female meiosis, this problem is circumvented in the following way: crossovers are restricted to form only a single chiasma per bivalent, which then triggers the redistribution of proteins along the bivalent axis, creating subdomains that define the region of cohesin removal and protection during meiosis I (ref. 28). Kinetochore components uniformly coat each half bivalent but are excluded from the midbivalent region. The chromosomes are embedded in massive microtubule bundles, and during anaphase I homologous chromosomes are segregated to the poles by microtubule forces pushing from the midbivalent regions towards the poles^29^,^30^. Sister chromatids remain attached via the other bivalent axes and are separated during the second meiotic division^28^,^29^,^31^.

Some other organisms with holocentric chromosomes, including plants (for example, *Luzula campestris* or *Cuscuta babylonica*), may circumvent the problem of meiosis by a different strategy. They were reported to display a diploid number of individualized chromatids at prophase II, indicating complete loss of sister chromatid cohesion in meiosis I. Accordingly, several authors have suggested that sister chromatids may segregate at anaphase I leading to the concept of inverted meiosis in which the order of reductional and equational division is inverted and separation of homologous non-sister chromatids follows sister chromatid segregation^23^,^32^,^33^,^34^,^35^,^36^,^37^,^38^,^39^. For successful generation of haploid generative cells via inverted meiosis, at least three requirements have to be met: (1) bipolar orientation of sister kinetochores and their attachment to microtubules from opposite spindle poles in meiosis I (amphitelic attachment); (2) segregation of sister chromatids to opposite poles in anaphase I (equational division); and (3) a mechanism to align and distribute homologous non-sister chromatids during the second meiotic division. So far, the occurrence of inverted meiosis has received strongest support by studies of a mealybug species (*Hemiptera*) in which a diploid individual with a heteromorphic chromosome pair was analysed^33^,^38^. Further evidence for inverted meiosis and also for its occurrence in the plant kingdom is still absent.

Here we present an in-depth analysis on the meiotic behaviour of two Cyperaceae species with holocentric chromosomes, *Rhynchospora pubera* (*n*=5) and *R. tenuis* (*n*=2). Our data support the occurrence of inverted meiosis in plants based on the observation that sister chromatids display amphitelic attachment to the spindle, that sister chromatids are subsequently separated during meiosis I and that homologous non-sister chromatids display mostly regular disjunction in anaphase II. Furthermore, the availability of a *R. pubera* individual with a heteromorphic chromosome pair allows the non-ambiguous reconstruction of the inverted meiotic sequence. The analyses of both species are complementary, since *R. pubera* displays chiasmatic meiosis, which is most commonly found among plants with holocentric chromosomes, while *R. tenuis* represents an exceptional case with achiasmatic meiosis.

## Results

### Chiasmatic meiosis of *R. pubera*

**Figure 1:**
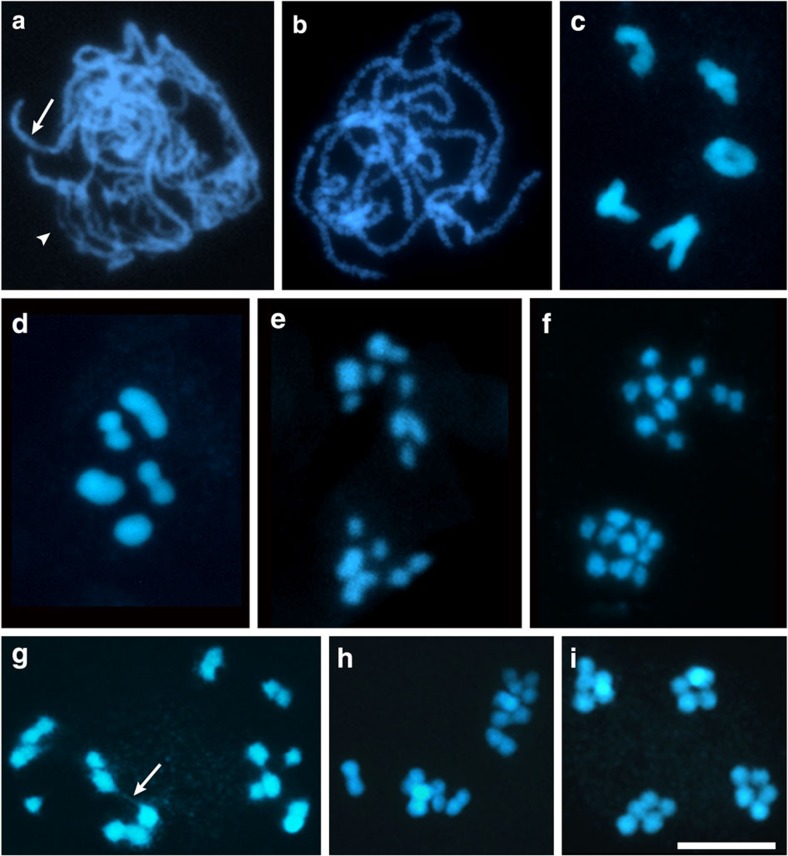
*R. pubera* meiosis. DAPI images representing different stages of meiosis. (**a**) Zygotene with paired (arrow) and unpaired (arrowhead) chromosomal regions. (**b**) Pachytene with completely paired chromosomes. (**c**) Diakinesis with one ring bivalent and four bivalents with one terminal/subterminal chiasma. (**d**) Metaphase I. (**e**) Anaphase I showing some individualized chromatids being pulled to either pole. (**f**) Prophase II with 10 individualized chromatids at each pole. (**g**) Prophase II showing chromatid pairs visibly connected by chromatin threads (arrow). (**h**) Metaphase II. (**i**) Late anaphase II. Size bar corresponds to 10 μm.

An overview of *R. pubera* meiosis has been described previously^24^. Briefly, in early prophase I chromosomes pair and synapse, inferred by the change in thickness of the filamentous chromosomes in the transition from zygotene to pachytene (Fig. 1a,b). Following condensation, five bivalents were observed in diakinesis (Fig. 1c), bearing most frequently one chiasma (71.3%, *n*=1,379 diakinesis and metaphase bivalents). Bivalents with two chiasmata and univalents were also observed, but with lower frequencies (25.2% and 3.5%, respectively; Supplementary Fig. 1a,b). Chiasmata were mostly positioned close to the chromosome ends (*n*=342 diakinesis bivalents). We infer that several aspects of *R. pubera* are similar to meiotic progression of other plants (for example, *Arabidopsis* or maize). First, we see deposition of the axial element protein ASY1 (refs 40, 41), indicating conservation of axis architecture (Supplementary Fig. 1d). Second, we observed RAD51 foci in prophase I cells (but not in mitotic cells), indicating that meiotic DSBs are formed and processed^42^ (Supplementary Fig. 1e). Third, despite the fact that we observe numerous RAD51 foci (indicative for numerous DSBs), only very few crossovers are formed. The small number of crossovers per bivalent and their preferential terminal localization in case of bivalents with two crossovers indicate strong CO interference. At metaphase I, bivalents with a single chiasma typically assumed a dumbbell shape (Fig. 1d). At anaphase I, mostly individualized chromatids are pulled to the poles (Fig. 1e). During anaphase I and prophase II, many cells contain (up to 10) individualized chromatids, instead of the five pairs of chromatids that would be expected for a canonical meiosis (Fig. 1f). In many cases thin chromatin threads, connecting the individualized 4',6-diamidino-2-phenylindole (DAPI)-stained bodies (in some instances more than two are connected), can be observed (Fig. 1g). At metaphase II, chromatids associate into pairs (Fig. 1h) and segregation of five chromatids to each pole occurs in anaphase II (Fig. 1i). These observations were intriguing, since they suggested that meiosis in *R. pubera* does not follow the canonical meiotic steps but rather that sister chromatids are separated in anaphase I and that the thin chromatin threads represent connections of homologous non-sister chromatids.

**Figure 2:**
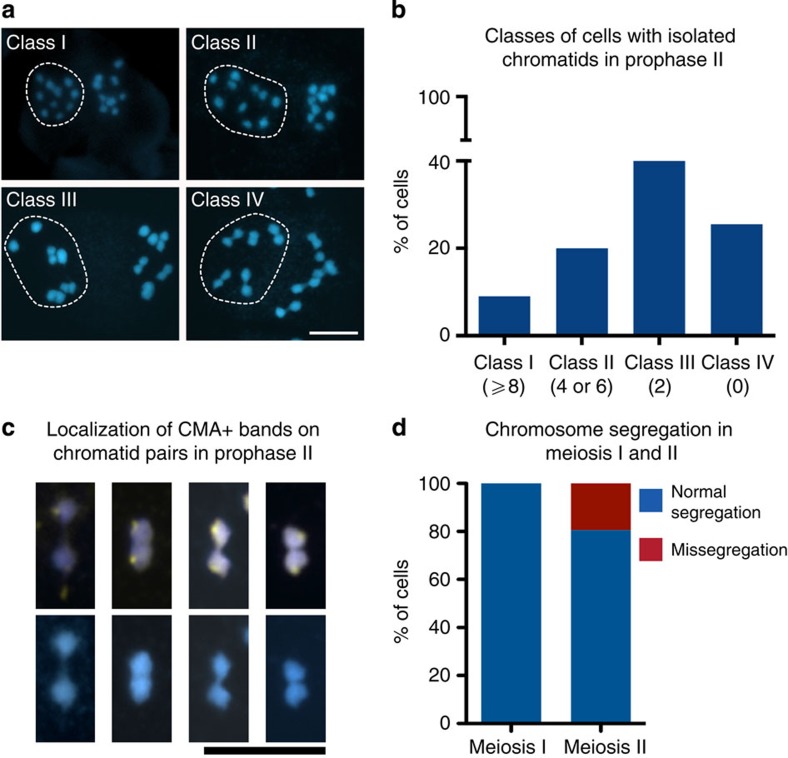
Association of chromatids in meiosis II of *R. pubera*. (**a**) Different classes of cells with individualized chromatids in prophase II. Class I comprises cells with eight or more individualized chromatids, Class II cells have four or six, class III have two and class IV represents cells without individualized chromatids. Daughter cells exemplifying each class are highlighted. **(b**) Frequency of the different classes of cells with isolated chromatids during prophase II (*n*=55). (**c**) Pairs of chromatids of prophase II cells connected with a thin DAPI (blue)-stained thread or in close association. CMA staining (yellow) indicates regions of 45S rDNA. (**d**) Quantification of chromosome mis-segregation in meiosis I and II (*n*=37 and 36 cells, respectively). Size bars correspond to 10 μm.

We quantified our observations and found that 9% of all prophase II cells had eight or more isolated chromatids (class I), 20% had four to six isolated chromatids (class II), 45.5% had only two isolated chromatids (class III) and 25.5% had no isolated chromatids (class IV, *n*=55; Fig. 2a,b). Chromatids were counted as pairs when they were in close proximity or at least connected by a chromatin thread (Fig. 2c). In all, 21.5% of all chromatids were not connected by threads, 10.7% were connected by threads and 67.8% appeared in close proximity (*n*=270 pairs of chromatids). We were interested whether chromatid association would be directed by 45S rDNA repeat regions (to be found on 3 of the 5 chromosomes of *R. pubera*)^43^ as described for the sex chromosome of *D. melanogaster*. We performed chromomycin A3 (CMA) staining that labels the 45S rDNA clusters in many plant species^43^,^44^,^45^ and also in *R. pubera* (Supplementary Fig. 2a). It is evident that 45S rDNA is not involved in mediating the interactions of chromatids during prophase II in *R. pubera*, since in none of the observed cases (*n*=63) the parts of the chromatids that face each other or the chromatin threads are associated with CMA staining (Fig. 2c). Since the rDNA clusters are located terminally on the *R. pubera* chromosomes^46^, terminal associations would either involve the 45S rDNA regions, which is not the case, or position them at the other end of the paired or associated chromatids. Interestingly, during metaphase I, those rod bivalents with 45S rDNA or CMA labelling were always arranged with the 45S rDNA clusters pointing outwards (*n*=141; Supplementary Fig. 2b).

During prophase II, chromatids were predominantly connected to a partner (Fig. 2a–c), yet some chromatids were clearly not. As outlined above, chromatids (either sister chromatids, in case of a canonical meiosis, or homologous non-sister chromatids, in case of an inverted meiosis) have to be connected at metaphase II to ensure regular disjunction. Since we observed some univalents in meiosis I and isolated chromatids in prophase II, we investigated mis-segregation in meiosis I and meiosis II. Indeed, we observed that 19.5% (*n*=36) of all meiosis II products had incorrect numbers of chromosomes (Fig. 2d; Supplementary Fig. 2c); however, none of the analysed cells showed mis-segregation during meiosis I (*n*=37). We assume that segregation of chromatids during meiosis II not only yielded ~80% of products with the correct number of chromatids but also with the correct set, since we only observed limited pollen abortion in anthers (Supplementary Fig. 2d). This indicates that (a) the occasional univalents (observed in diakinesis) do not lead to unbalanced chromatid numbers in telophase I/prophase II and that (b) those isolated DAPI-stained bodies observed in prophase II may actually represent single sister chromatids, which separated during meiosis I from their respective sister, but have failed to connect to a homologous non-sister chromatid and are therefore mis-segregating in meiosis II.

**Figure 3:**
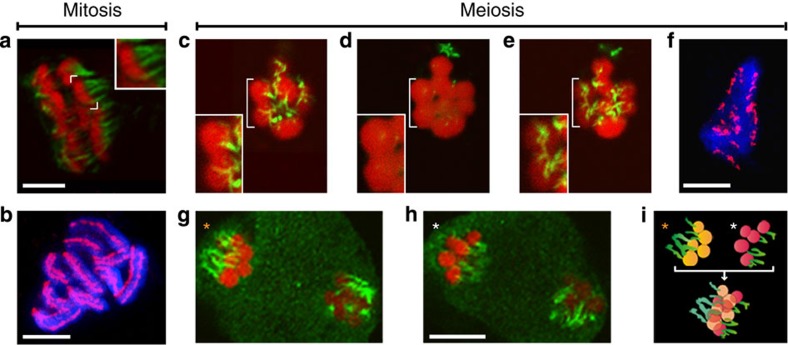
Microtubule attachment on chromosomes of *R. pubera*. (**a**) Mitotic metaphase with one chromosome highlighted in the upper corner. Note the multiple sites of microtubule attachment. (**b**) Mitotic metaphase with chromosomes labelled with an antibody directed against CENH3, which localizes in two parallel lines along each chromosome. (**c**–**e**) Polar view of meiotic metaphase I with one bivalent highlighted. The same cell is viewed in an upper focal plane (**c**), a medium focal plane (**d**) and a lower focal plane (**e**). (**f**) Chromosomes at meiotic metaphase I labelled with an antibody directed against CENH3, which localizes in distinct patches along both sides of each bivalent. (**g**–**h**) Metaphase II. Upper (**g**) and lower (**h**) focal planes of the same cell. (**i**) Scheme of orientation of metaphase chromosomes marked with * in **g**,**h** representing the five chromatids attached to microtubules from each spindle pole and an overlay showing the bipolar orientation. (**a**,**c**–**e**,**g**,**h**) Chromosomes in red, microtubules in green. (**b**,**f**) Chromosomes in blue, CENH3 in magenta. Size bars correspond to 5 μm.

While our observations depict meiotic peculiarities not described for other organisms so far, they do not provide direct evidence for an equational first meiotic division and a reductional second division. To further investigate the nature of *R. pubera* meiosis, we analysed the meiotic spindle and its attachment to chromosomes since we envisaged that knowing the mode of spindle attachment to meiotic chromosomes would allow drawing conclusions about chromosome/chromatid segregation during meiosis I. In mitotic cells (Fig. 3a; Supplementary Movie 1), 10 holocentric chromosomes align along the metaphase axis and each is attached to the spindle at various sites in a bipolar manner (amphitelic attachment). The kinetochores can be visualized all along the chromosome as parallel axes of CENH3 labelling (Fig. 3b). In meiosis I, the five bivalents are highly condensed and (especially well visible in dumbbell/rod bivalents) are aligned on the metaphase plate with their longitudinal axes perpendicular to the spindle. Microtubules are attached to the central regions and on both sides of each half bivalent, resulting in multiple spindle attachment regions per bivalent (*n*=20; Fig. 3c–e; Supplementary Movies 2 and 3). Each half of such a (dumbbell) bivalent is assumed to consist of two sister chromatids (see Supplementary Fig. 3a and Fig. 7 for schematic representations). This assumption is based on observations in *C. elegans*, which shows bivalents with a dumbbell shape bearing a single subterminal chiasma, resembling the bivalents of *R. pubera*^27^. To confirm the localization of microtubules in the central region of the ‘half bivalents’, the 45S rDNA clusters, localized at the termini of three chromosomes in *R. pubera*^43^, were visualized with fluorescent *in situ* hybridization (FISH; Supplementary Fig. 2b). As mentioned above, in all dumbbell-shaped bivalents containing the 45S rDNA cluster, these regions were localized at the chromosome termini opposite to the associated regions and not at the central region where microtubules attach. The microtubule immunostaining also revealed that there are several microtubule attachment sites in metaphase I chromosomes, similar to the less condensed mitotic chromosomes. These observations correlate well with the localization of CENH3 on the highly condensed metaphase I bivalents, showing multiple patches of labelling in two parallel lines (Fig. 3f). It is interesting to note that the distribution of the mitotic centromere marker H2AThr120ph gives a diffuse staining around the meiotic metaphase chromosomes, while in mitotic chromosomes its localization resembles CENH3, indicating substantial reorganization of the meiotic centromeres (Supplementary Fig. 3b). In metaphase II, each chromatid was associated with microtubules from one cell pole (*n*=48; Fig. 3g–i; Supplementary Movie 4).

In comparison with a regular meiosis, as described for the holocentric organism *C. elegans*, multiple differences have been identified in *R. pubera*. In *C. elegans* the meiotic spindle forms microtubule bundles that surround the bivalents (female meiosis) or shows terminal associations to bivalents (male meiosis) at metaphase I, and, importantly, bivalents orient themselves with the long axis parallel to the spindle^29^. In *R. pubera*, bivalents are oriented with the longer axis perpendicular to the spindle and they appear to have several microtubule attachment sites with kinetochores of sister chromatids attached independently to microtubules emanating from opposite poles (amphitelic attachment). At anaphase I, *C. elegans* homologues are separated, while in *R. pubera* sister chromatids are separated from each other and pulled to different poles. At this stage, sister chromatids of *C. elegans* are still held together by cohesins and, in contrast, the non-sister chromatids of *R. pubera* appear individualized, with some being connected with thin chromatin threads. Importantly, the diploid number of DAPI-stained bodies can clearly be visualized in each half of the dyad in prophase II, following the expectations of sister separation during meiosis I in *R. pubera*. In *C. elegans* at metaphase II/anaphase I, sister chromatids align and are subsequently separated. In *R. pubera*, chromatids associate with the help of an unknown mechanism and subsequently undergo disjunction during anaphase II. These observations are in strong favour of sister separation during meiosis I in *R. pubera* (Supplementary Fig. 3).

**Figure 4:**
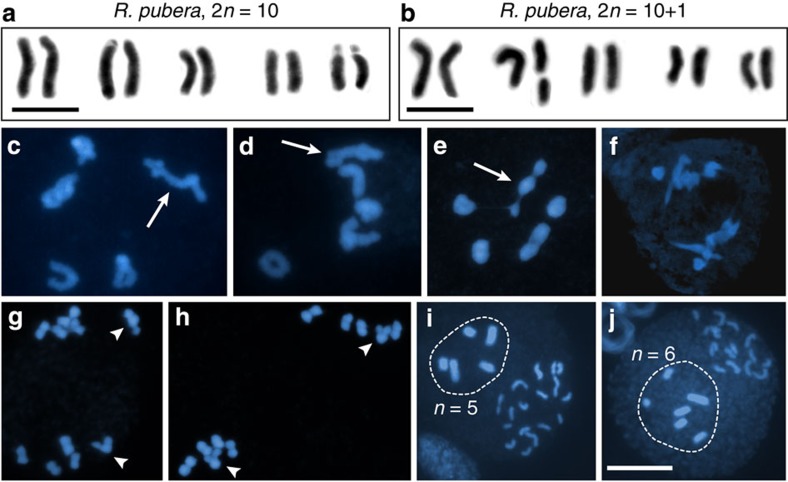
Meiosis of *R. pubera* (2*n*=10+1). (**a**) Karyotype of *R. pubera* 2*n*=10. (**b**) Karyotype of *R. pubera* 2*n*=10+1. (**c**–**j**) DAPI images representing different stages of meiosis. (**c**,**d**) Cells in diakinesis showing the pairing behaviour of the heteromorphic bivalent (arrows). (**e**) Metaphase I. Arrow points to heteromorphic bivalent. (**f**) Anaphase I. (**g**,**h**) Cells in prophase II/metaphase II showing equational segregation of chromatids of the heteromorphic bivalent (arrowheads). (**i**,**j**) Microsporogenesis showing examples of pseudomonads in which the nucleus with (**i**) *n*=5 or (**j**) *n*=6 (highlighted) is centrally positioned and will give rise to a pollen grain. Size bars correspond to 10 μm.

We gained further evidence of the independent orientation and separation of sister kinetochores in meiosis I by analysing chromosome segregation of a *R. pubera* individual with an apparent chromosome breakage in chromosome 2 (Fig. 4a,b). As observed for other species^47^, fragments of holocentric chromosomes can acquire new telomeric sequences and be stably transmitted in *R. pubera* as well. This heteromorphic chromosome pair provided an ideal test system for the analysis of chromosome segregation during meiosis. In case sister chromatids are separated during the first meiotic division (equational division), each of the two cells of the resulting dyad should contain the same number of DAPI-stained bodies (chromatids and chromatid fragments). In contrast, if homologous chromosomes are separated then the number of DAPI-stained bodies (chromatids and chromatid fragments) is not expected to be the same, with one cell of the dyad receiving the intact chromosome 2 and the other one the two parts of the fragmented chromosome 2 (A schema for the two different scenarios can be found in Supplementary Fig. 4). The plants were phenotypically normal and fertile; however, during prophase of meiosis I a heteromorphic bivalent was visible. The heteromorphic bivalent formed terminal chiasmata involving both broken parts (*n*=45; a non-associated chromatid fragment was never observed), visible during diakinesis (Fig. 4c,d) and metaphase I (Fig. 4e). All post anaphase I and prophase II cells analysed (*n*=24) clearly showed mirror images of the heteromorphic chromosome pair, demonstrating that meiosis I in *R. pubera* is indeed equational (Fig. 4f–h). In *R. pubera* three of the four microspores generated are degraded^48^ and the individual plant with the broken chromosome 2 seems not to be different in this respect, showing pollen grains with five or six DAPI-stained bodies (Fig. 4i,j).

### Achiasmatic meiosis of *R. tenuis*

**Figure 5:**
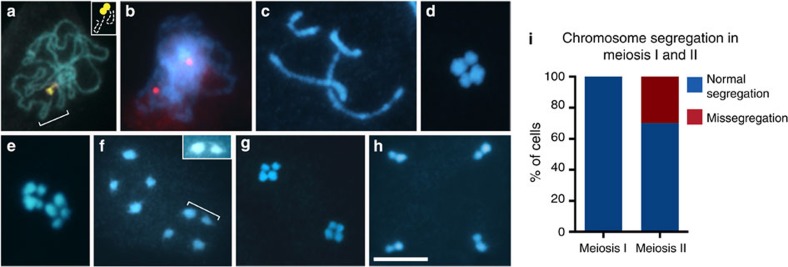
Meiosis of *R. tenuis*. DAPI Images representing different stages of meiosis. (**a**) Zygotene showing end-to-end association of chromosomes carrying the CMA^+^ bands (yellow). (**b**) Zygotene showing unpaired 5S rDNA sites (in red). (**c**) Diplotene/diakinesis with four univalents. (**d**) Metaphase I with univalents organized in a cross-like shape. (**e**) Lateral view of anaphase I. (**f**) Prophase II. The inset shows the highlighted chromatids with adjusted brightness and contrast to enhance visibility of chromatin threads. (**g**) Metaphase II. (**h**) Late anaphase II. (**i**) Quantification of chromosome mis-segregation in meiosis I and II (*n*=107 and 80 cells, respectively). Size bar corresponds to 10 μm.

*R. tenuis* (*n*=2) is closely related to *R. pubera*, and its analysis provides further support for the occurrence of inverted meiosis in the *Cyperaceae* family. An advantage for analysis is that the two chromosomes differ in their size and can easily be distinguished. To analyse chromosome pairing and synapsis during *R. tenuis* early prophase, the small chromosome pair was specifically labelled with CMA (anticipated to label the nucleolus-organizing region^45^,^49^). The second chromosome pair was identified by *in situ* hybridization of the 5S rDNA cluster, localizing to the interstitial region of the chromosome lacking the CMA^+^ signal^46^. The CMA-chromosome primarily displayed end-to-end associations (71%, *n*=90) during early prophase I (Fig. 5a), while lateral alignment, indicative of pairing, was less often observed. The 5S rDNA marker on the second chromosome pair appeared unpaired in 80% of the cells analysed (zygotene/pachytene stage; *n*=33) suggesting the absence of pairing also for this chromosome (Fig. 5b).

Interestingly, other aspects of meiosis seemed conserved, since deposition of the meiosis-specific axial element protein ASY1 indicates that the overall meiotic chromosome organization is similar compared with other plant species including *R. pubera* (Supplementary Fig. 5a). We also observed RAD51 foci in meiotic cells (but not in mitotic cells) of *R. tenuis* and believe that they indicate programmed meiotic DSB formation and processing (Supplementary Fig. 5b). Yet, when chromosomes condensed further in diplotene/diakinesis four univalents could be clearly distinguished and chiasmata were never observed (*n*=210; Fig. 5c). This indicates that the DSBs formed earlier during prophase are repaired via non-crossover pathways. At metaphase I, these four non-paired univalents aligned at the equatorial plane forming a cross-like figure (Fig. 5d). In anaphase I, four chromatids (two of each size) are pulled to each pole indicating segregation of sister chromatids (*n*=107; Fig. 5e; Supplementary Fig. 6). It is interesting to note that in prophase II the four chromatids are associated via thin chromatin threads, forming connections between two or more chromatids (Fig. 5f). At metaphase II the four chromatids in each part of the dyad were again arranged in a cross-like configuration (Fig. 5g) and separated in anaphase II with two chromatids of different size segregating (Fig. 5h) to the respective poles.

While segregation of sister chromatids appears error-free during the first meiotic division, 30% of the post-anaphase II stages (*n*=80) had an irregular genomic content (Fig. 5i; Supplementary Fig. 5c). It is interesting to note that this mostly concerned the type of chromatids (for example, two large or two small chromatids together; 25%) and only in few cases aneuploidy (5%). In agreement with the meiosis II segregation defects, we also observed some nonviable pollens (Supplementary Fig. 5d). Taken together, this indicates that sister chromatid cohesion in *R. tenuis* is completely lost during the first, achiasmatic meiotic division and, furthermore, that during the second meiotic division homologous non-sister chromatids have only a partially functional compensatory mechanism to enhance regular disjunction.

**Figure 6:**
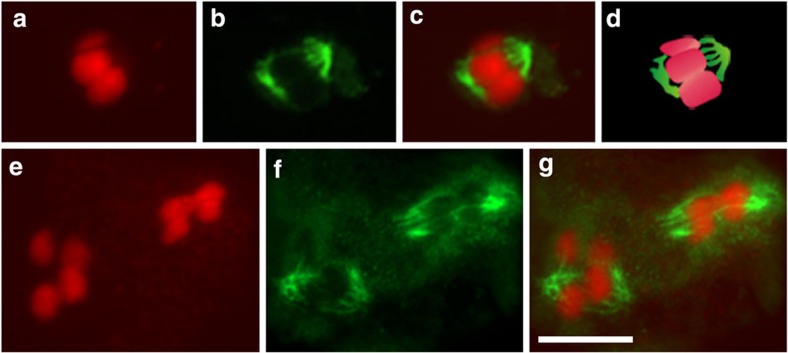
Microtubule attachment in *R. tenuis* meiosis. (**a**–**c**) Lateral view of metaphase I chromosomes (**a**, red) showing microtubules (**b**, green) attached to both sides of each univalent. Only three out of four univalents are visible. (**d**) Scheme of the overlay shown in **c**. (**e**–**g**) Early anaphase II with chromatids (**e**, red) individually attached to microtubules **(f**, green). (**g**) Overlay of **e**,**f.** Size bar corresponds to 5 μm.

To further corroborate the finding of an equational nature during the first meiotic division in *R. tenuis*, we performed immunostaining of microtubules. In metaphase I, each univalent associates with microtubules emanating from opposite poles of the cell, thereby displaying an amphitelic attachment to the spindle (*n*=22; Fig. 6a–d). During metaphase II (*n*=142) and anaphase II (*n*=54; Fig. 6e–g), each chromatid appears to be associated with microtubules from only one cell pole. The described results are intriguing since the predictions for achiasmatic meiosis embedded in either a regular or inverted pathway are very different. In the canonical meiotic pathway, non-paired univalents are expected to be randomly distributed at anaphase I. In contrast, in case of inverted meiosis, sister chromatids of each univalent are expected to become individually attached to the spindle and to be separated during anaphase I. Therefore, the expectation for inverted meiosis is, to see four chromatids in both parts of the resulting dyad and also error-free, reliable disjunction. Indeed, these expectations are always met (*n*=107). In metaphase II, chromatids align and subsequently undergo disjunction during anaphase II. In the case of achiasmatic meiosis, following the canonical pathway, the second meiotic division is expected to resemble an equational, error-free division. In contrast, the second division of an inverted meiosis is expected to face the problem of distributing homologous non-sister chromatids. Depending on the accuracy of a hypothetical mechanism to promote regular disjunction, errors during chromatid disjunction are expected in the second meiotic division of an inverted meiosis. Indeed, about 30% of all meiotic products of *R. tenuis* show irregularities. These results strongly support the claim of inverted meiotic events in *R. tenuis* (Supplementary Fig. 6).

## Discussion

The occurrence of a diploid number of individualized chromatids in prophase II has been the principal indication for inverted meiosis in several species dating from very early studies on meiosis of holocentric chromosomes^32^,^34^,^37^,^38^. However, this deviation from the canonical progression through meiosis could be ascribed to either separation of sister chromatids in anaphase I (equational division) or to disjunction of homologous chromosomes followed by loss of sister chromatid cohesion (reductional division with premature cohesion loss). To our understanding, and as outlined above, genuine inverted meiosis has to meet at least the criteria of: (1) bipolar orientation of sister chromatids and their attachment to opposite spindle poles in meiosis I; (2) segregation of sister chromatids to opposite poles in anaphase I (equational division); and (3) a mechanism to align and distribute homologous non-sister chromatids during meiosis II.

Here we present robust evidence for inverted meiosis in two related plant species of the *Cyperaceae* family, *R. pubera* and *R. tenuis*. We found that *R. tenuis* separates sister chromatids equationally during the first meiotic division. Meiosis in *R. tenuis* is achiasmatic, which means that the homologous chromosomes do not exchange genetic material and that they do not become connected during meiotic prophase I via chiasmata. The connection of sister chromatids is apparently completely lost at the end of meiosis I, yet chromatids are not distributed randomly during anaphase II.

Does meiosis in *R. tenuis* fulfil all criteria to be defined as ‘inverted meiosis’? Certainly, sister chromatids have a bipolar orientation and are attached to opposite spindle poles (amphitelic attachment) in meiosis I. Furthermore, sister chromatid cohesion at the onset of anaphase I is lost and sister chromatids segregate to opposite poles. It should be noted that the haploid genome complement of *R. tenuis* comprises only two chromatids^50^. In this sense, the extreme reduction of chromosome number could be seen as an adaptation to the inverted, achiasmatic meiotic sequence with a good stochastic chance to end up with the correct set of chromosomes in a generative cell. Interestingly, we observed that 70% of all meiosis II products contained the correct set, which is well above the expected 25% if chromatids would be distributed randomly in anaphase II. In the light of the given numbers, the thin chromatin threads visible in *R. tenuis* prophase II, which connect chromatids with each other, could be instrumental to support regular disjunction. Although we do not have indication of their sequence composition, these threads could be of heterochromatic nature and resemble those found to connect achiasmatic chromosomes during meiosis I in *D. melanogaster.* It is important to note that in *R. tenuis* these connections cannot be the remnants of recombination, since this species displays achiasmatic meiosis. Pradillo *et al.*^51^ suggest that SC components may enhance regular disjunction of univalents during meiosis I in *Arabidopsis* mutants with impaired DSB formation or interhomologue bias. Certainly one can also envisage that in *R. pubera* and *R. tenuis*, SC-related proteins may support regular segregation of homologous non-sister chromatids during meiosis II.

**Figure 7:**
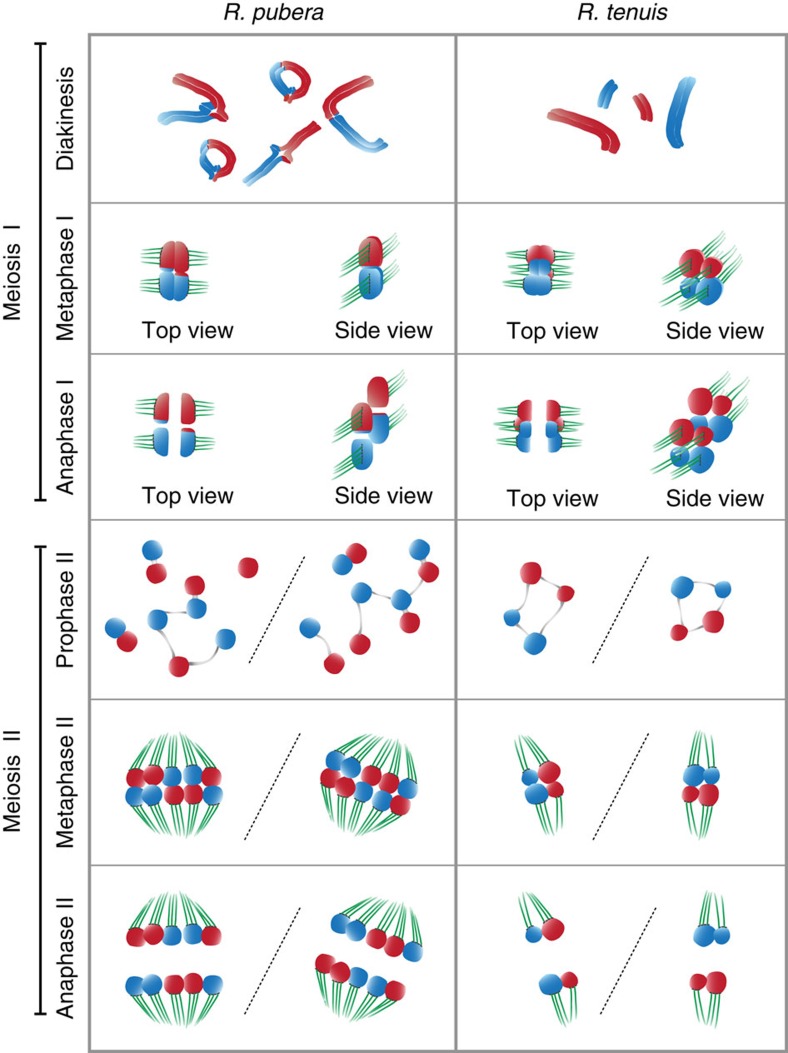
Model for meiotic events in *R. pubera* and *R. tenuis*. During meiosis I chromosomes of *R. pubera* recombine, pair and bivalents with chiasmata become visible in diakinesis. In contrast, chromosomes of *R. tenuis* do not form chiasmata and univalents become visible. In metaphase I (only one, rod-shaped bivalent of *R. pubera* is shown; all four chromosomes of *R. tenuis* are shown), chromosomes align at the metaphase plate and sister chromatid show amphitelic attachment to the spindle. In anaphase I (only one, rod-shaped bivalent of *R. pubera* is shown; all four chromosomes of *R. tenuis* are shown), sister chromatids are separated from each other and pulled to different poles. For prophase II, each half of the dyad is shown. While chromatids of *R. pubera* appear mostly in pairs, with some chromatids being connected with thin chromatin threads, all four chromatids of *R. tenuis* appear interconnected by thin chromatin threads. In metaphase II, chromatids align and subsequently undergo regular disjunction during anaphase II. Note, the model idealizes regular disjunction in meiosis II but actually 19.5 and 30% of all meiotic products of *R. pubera* and *R. tenuis*, respectively, show irregularities. Refer to text for further details. Parental chromosomes/chromatids are in red and blue, the spindle in green and chromatin threads in grey.

We believe that the chromatin threads are part of a mechanism to associate the four chromatids during prophase II and align them during metaphase II. Even though a specific mechanism to link corresponding homologous non-sister chromatids may not be in place, the presumably nonspecific association of the chromatids would allow balancing of the force exerted by the metaphase II spindle. Since the chromatids in *R. tenuis* are of very different size and would, therefore, according to their holocentric nature accommodate more or less microtubule attachment sites, we envisage a model of balanced spindle forces only in case one large and one small chromosome is connected to each spindle pole, thereby promoting regular chromatid disjunction (Fig. 7). In fact, with the single assumption that the spindle is organized such that there is indeed a preference for only two chromatids connecting to one pole, then even random segregation would result in a correct genome complement in ~66% of all meiosis II division events. This number is in good agreement with the experimental data.

Meiotic progression is more complex in *R. pubera*. First, *R. pubera* has five chromosomes and homologous chromosomes pair and form chiasmata in meiosis I. Interestingly, univalents have been observed infrequently during prophase I; yet no unequal segregation was observed during anaphase I. This is intriguing as it suggests that, similar to cases described earlier^52^,^53^,^54^, also in *R. pubera* univalents are segregated equationally during meiosis I. Furthermore, during anaphase I, 10 isolated DAPI-stained bodies (a diploid number of isolated chromatids) moved towards each pole. Together, this indicates loss of sister chromatid cohesion and equational division.

Further evidence to support the idea of sister chromatid segregation during meiosis I is the observation that in *R. pubera* each bivalent has multiple microtubule attachment sites. These sites are in the central region of each half bivalent, with sister chromatids being attached to spindles from different poles (Fig. 7). This amphitelic attachment is one of the prerequisites for inverted meiosis. It would certainly be ideal to distinguish sister chromatids and homologous non-sister chromatids directly (for example, with FISH probes specific for only one of the two homologues or LacO arrays inserted only in one of the two homologues). Unfortunately, advanced tools are not available for the two non-model plants investigated in this study. Nonetheless, the availability of a plant with a heteromorphic chromosome pair allowed to nonambiguously define segregation of sister chromatids during meiosis I in *R. pubera*, similar to earlier studies in the homopteran *Planococcus citri*^38^.

Does meiosis in *R. pubera* fulfil all criteria to be defined as ‘inverted meiosis’? As outlined above, the kinetic activity centres of the sister chromatids are in bipolar orientation. During anaphase I, sister chromatid cohesion seems to be lost and isolated chromatids segregate to opposite poles. In this sense, two of the three criteria for genuine inverted meiosis are satisfied. The question how homologous non-sister chromatids find each other during meiosis II to promote regular disjunction remains open.

In *R. pubera* prophase II, the chromatids resulting from anaphase I separation appear mostly as pairs or with DAPI-stained threads connecting them. Some of the chromatid pairs are at a greater distance to each other, connected by a thin chromatin thread and, in some cases, individualized chromatids are connected to more than one chromatids. As in the case for *R. tenuis*, we believe that these chromatin threads are instrumental for associating (homologous) non-sister chromatids in preparation for the second meiotic division.

These threads could be heterochromatin; however, in contrast to *D. melanogaster*, which employs in some instances heterochromatic 45S rDNA repeat containing regions to connect achiasmatic chromosomes during meiosis I (ref. 16), the *R. pubera* 45S rDNA regions are not involved in these interactions. We do not believe that these threads are connected by maintained cohesion proteins, since bivalents with two crossovers (25% of all bivalents in *R. pubera*) would eventually have one sister chromatid connected to two different non-sister chromatids that would subsequently interfere with chromosome segregation; however, anaphase bridges have not been observed (*n*=30). The idea of chromatin connections between non-sister chromatids finds further support by the results presented above for the achiasmatic meiosis in the closely related plant *R. tenuis* and by a parallel study performed in the holocentric plant *Luzula elegans*^55^. It appears unlikely that different solutions to cope with holocentricity have evolved in these closely related species. In case of *R. pubera*, we envisage a similar model as outlined for *R. tenuis*, with the addition that during prophase II chromatin connections may preferentially form between homologous non-sister chromatids since they originated from the same bivalent and were therefore in closer proximity.

Nevertheless, alternative ways to ensure proper chromosome segregation during meiosis emerged in other organisms with holocentric chromosomes. For instance, in *C. elegans* the chiasma position defines the orientation of bivalents to the spindle and thus how they attach to spindle microtubules^29^,^31^ as also the regions for cohesin maintainance in meiosis I (ref. 28). Importantly, regions of sister chromatid cohesion maintenance also display synthetic attachment to the spindle in *C. elegans*. It appears that *R. pubera* and *R. tenuis* have adapted a different mode to deal with the holocentric nature of chromosomes during meiosis characterized by (I) amphitelic sister chromatid attachment in metaphase I, by (II) equational sister chromatid separation in anaphase I and (III) by employing a chromatin thread-mediated mechanism in prophase II to associate (homologous) non-sister chromatids for regular disjunction in meiosis II. Altogether, these meiotic alterations can be framed under the term ‘inverted meiosis’.

## Methods

### Plant material

Individuals of *R. tenuis* were collected in Porto de Galinhas (Ipojuca, PE, Brazil) and individuals of *R. pubera* in Mata de Dois Irmãos (Recife, PE, Brazil). Both species were either cultivated in an open experimental garden or in a greenhouse. Vouchers are kept at the herbarium of the Federal University of Pernambuco.

### Slide preparation, DAPI staining and CMA/DAPI banding

Anthers were fixed in ethanol-acetic acid (3:1 v/v) and stored in fixative at −20 °C. Anthers were digested in 4% cellulase, 4% pectolyase and 4% cytohelicase at 37 °C for 4 h, and meiocytes were squashed in a drop of 60% acetic acid. Coverslips were removed after freezing in liquid nitrogen and slides were kept at −20 °C before use. Slides were stained with DAPI, 1 μg ml^−1^, for 30 min and mounted in glycerol/McIlvaine (1:1, v/v). The CMA/DAPI banding was performed as previously described^44^. For that, slides were aged for 3 days, stained with 0.5 mg ml^−1^ CMA for 1 h and restained with 2 μg ml^−1^ DAPI for 30 min more. Before analysis, slides were aged for 3 more days.

### Fluorescent *in situ* hybridization

FISH was performed as described in ref. 56 with some modifications. In brief, slides were treated with pepsin (0.1 mg ml^−1^) and denatured with 70% formamide for 10 min at 85 °C. Slides were then denatured once more together with the hybridization mix (50% (v/v) formamide, 10% (w/v) dextran sulfate, 2 × SSC, 10% (w/v) SDS, 2–5 ng μl^−1^ probe) for 10 min at 90 °C. After the stringency washes, slides were mounted with DAPI/Vectashield H-1000 (2 μg ml^−1^). The 45S rDNA was detected using the probe R2, a 6.5-kb fragment of an 18S–5.8S–25S rDNA repeat unit from *Arabidopsis thaliana*^57^, and the 5S rDNA was detected using the Rhy2 clone of *R. tenuis* as a probe^46^. The plasmid DNAs were isolated using the Plasmid Mini Kit (QIAGEN) and the 45S rDNA and 5S rDNA probes were labelled by nick translation (Invitrogen) with digoxigenin-11-dUTP (Roche Diagnostics) and Cy3-dUTP (GE Healthcare), respectively. The 45S rDNA probe was detected with sheep anti-digoxigenin fluorescein isothiocyanate (FITC) conjugate antibody (Roche) and amplified with rabbit anti-sheep FITC conjugate antibody (Dako).

### Immunostaining

The immunostaining for ASY1 and H2AThr120ph (ref. 58) was performed as described in^59^ with some modifications. Anthers were fixed in 4% paraformaldehyde in PBS buffer (1.3 M NaCl, 70 mM Na_2_HPO_4_, 30 mM NaH_2_PO_4_) for 40 min at room temperature (RT) and squashed in a drop of the same buffer. After coverslips were removed, slides were stained with DAPI/PBS (2 μg ml^−1^) for selection of appropriate meiotic stages. Slides were then washed with PBS and blocked with 3% bovine serum albumin (BSA) for 10 min at RT. The antibodies used were rabbit anti-AtASY1 (ref. 41), diluted 1:250 in blocking solution, and goat anti-rabbit IgG FITC conjugated (Sigma, no. F9887), diluted 1:300. Slides were counterstained and mounted with DAPI/Vectashield H-1000 (2 μg ml^−1^).

Immunostaining for RAD51 was performed as described in ref. 60 using a monoclonal mouse anti-Rad51 antibody (NeoMarkers, no. MS-988-P0), diluted 1:75, and goat anti-mouse IgG Alexa568 conjugated (Molecular Probes, no. A11031), diluted 1:300.

Immunolocalization of tubulin was performed as previously described^61^ with some modifications. Anthers were pretreated with 0.1 mM m-maleimidobenzoic acid N-hydroxysuccinimide ester for 15 min at RT and fixed with 4% paraformaldehyde in PEM buffer (50 mM PIPES, 5 mM MgSO_4_, 5 mM EGTA, 0.1% Triton X-100, pH 6.9) for 1 h at RT. Afterwards, anthers were squashed in the same buffer on a gelatin-coated slide. After coverslips were removed, slides were stained with DAPI/PEM (2 μg ml^−1^) and selected under a fluorescence microscope. The best slides were washed in PEM, digested with 1.4% β-glucuronidase for 30 min at RT and blocked with 3% BSA for 1 h at RT. The antibodies used were mouse anti-β-tubulin (Sigma, no. T9026), diluted 1:40, and rabbit anti-mouse IgG TRITC conjugated (DAKO), diluted 1:25, or goat anti-mouse IgG Alexa568 conjugated (Molecular Probes, no. A11031), diluted 1:300.

For anti-grassCENH3 (ref. 62) immunostaining anthers undergoing meiosis were covered with ice-cold 100% methanol, allowing cells to fix for 30 min at −20 °C. The fixative was then aspirated and anthers were rinsed three times in PBS for 5 min each. Pollen mother cells were squeezed out from the anthers and squashed in a drop of 1 × PBS. The coverslips were removed following freezing in liquid nitrogen. The immunostaining procedure was conducted as described above.

### Microscopy

Pictures of cells stained with DAPI or CMA/DAPI were taken with a Leica DMRB fluorescent microscope equipped with a Cohu digital camera and Leica Q-FISH programme. FISH pictures, together with RAD51 and ASY1 immunostaining pictures, were taken on a Carl Zeiss Axioplan 2 equipped with Photometrics Quantix camera and MetaMorph 7.1.4.0 software (Molecular Dynamics). Pictures of microtubule immunostaining were taken with the confocal microscope Carl Zeiss LSM 510 Meta using the LSM 510 programme. Image deconvolution was performed with the Auto Deblur 9.2.1 software (AutoQuant Imaging). Alternatively, pictures were taken with a Leica DM5500B microscope equipped with a deconvolution system and a Leica DFC345 FX camera. Further processing of images was made with HeliconFocus 5.0 and Adobe Photoshop CS4 softwares.

To analyse the substructures of immunosignals and chromatin beyond the classical Abbe/Raleigh limit at an optical resolution of ~120 nm (super resolution), structured illumination microscopy (SIM) was applied using a C-Apo × 63/1.2 W Korr objective of an Elyra microscope system and the software ZEN (Zeiss, Germany). Image stacks were captured separately for each fluorochrome using appropriate excitation and emission filters. Maximum intensity projections were generated from the stacks of optical SIM sections through the specimens by the ZEN software (three-dimensional-rendering based on SIM image stacks was carried out using the ZEN software).

## Additional information

**How to cite this article:** Cabral, G. *et al.* Chiasmatic and achiasmatic inverted meiosis of plants with holocentric chromosomes. *Nat. Commun.* 5:5070 doi: 10.1038/ncomms6070 (2014).

## Supplementary Material

Supplementary Information Supplementary Figures 1-6

Supplementary Movie 1 Confocal planes of mitotic metaphase of *Rhynchospora pubera*, corresponding to Fig. 3a. Chromosomes in red, tubulin in green.

Supplementary Movie 2 Confocal planes of meiotic metaphase I of *Rhynchospora pubera*,corresponding to Fig. 3c. Chromosomes in red, tubulin in green.

Supplementary Movie 3 3D view of meiotic metaphase I chromosomes and spindle of *Rhynchospora pubera*, corresponding to Fig. 3c. Chromosomes in red, tubulin in green

Supplementary Movie 4 Confocal planes of meiotic metaphase II of *Rhynchospora pubera*,corresponding to Fig. 3e. Chromosomes in red, tubulin in green.
