# Supplementary material for: Chiasmatic and achiasmatic inverted meiosis of plants with holocentric chromosomes
Source: Nat Commun. 2014 Oct 8;5:5070. doi: 10.1038/ncomms6070 (PMC4190664; doi:10.1038/ncomms6070)
Supplement: Supplementary Information — Supplementary Figures 1-6 [file ncomms6070-s1.pdf]

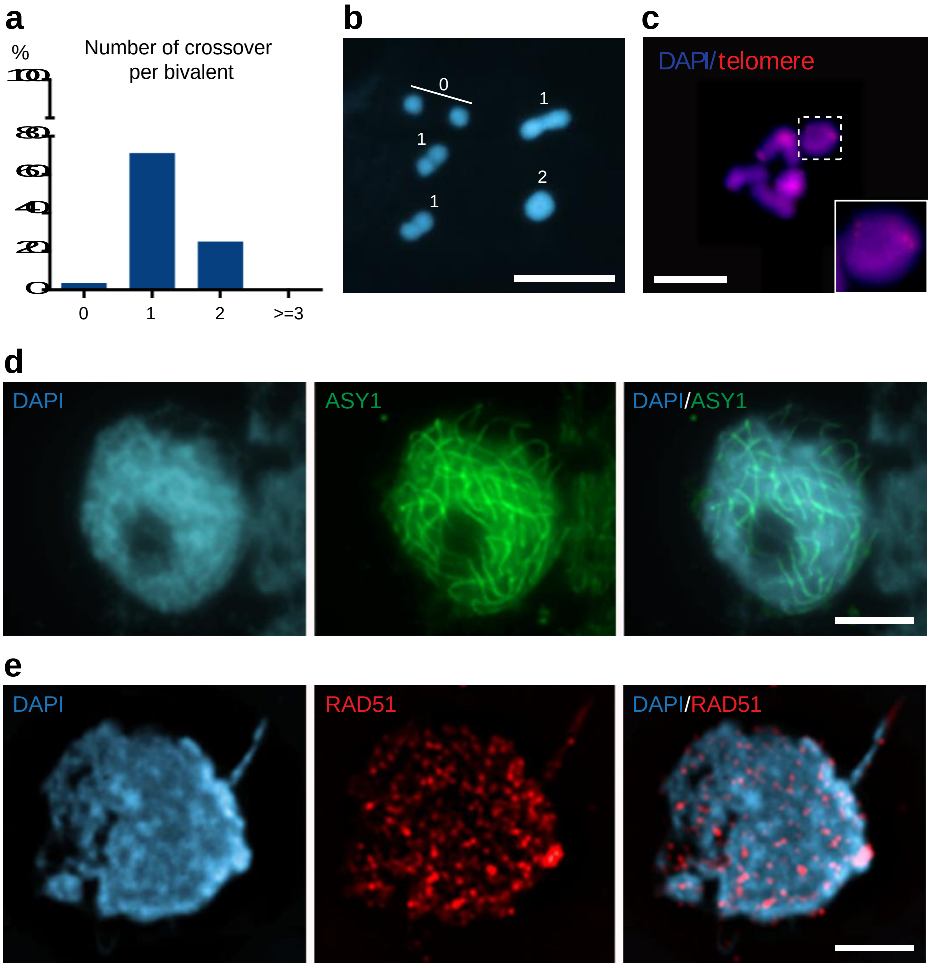

Supplementary Figure 1. Additional information on meiosis in *R. pubera*. a) The graph shows the distribution of chiasmata per bivalent/chromosome pair (n=1379) and the image in **b**) depicts a metaphase I cell showing one chromosome pair without a chiasma (0), three bivalents with one chiasma (1) and one bivalent with two chiasmata (2). **c**) Chiasmata have been observed at terminal positions. The panel highlights a ring bivalent with chiasmata located close to the telomere signals. **d**) Localization of ASY1 (green) in a leptotene nucleus indicates the formation of a meiotic chromosome axis. **e**) Leptotene nucleus with RAD51 (red) foci indicating the occurrence of DNA double strand breaks and subsequent processing. Size bars in (c), (d) and (e) correspond to 5µm and in (b) to 10µm.

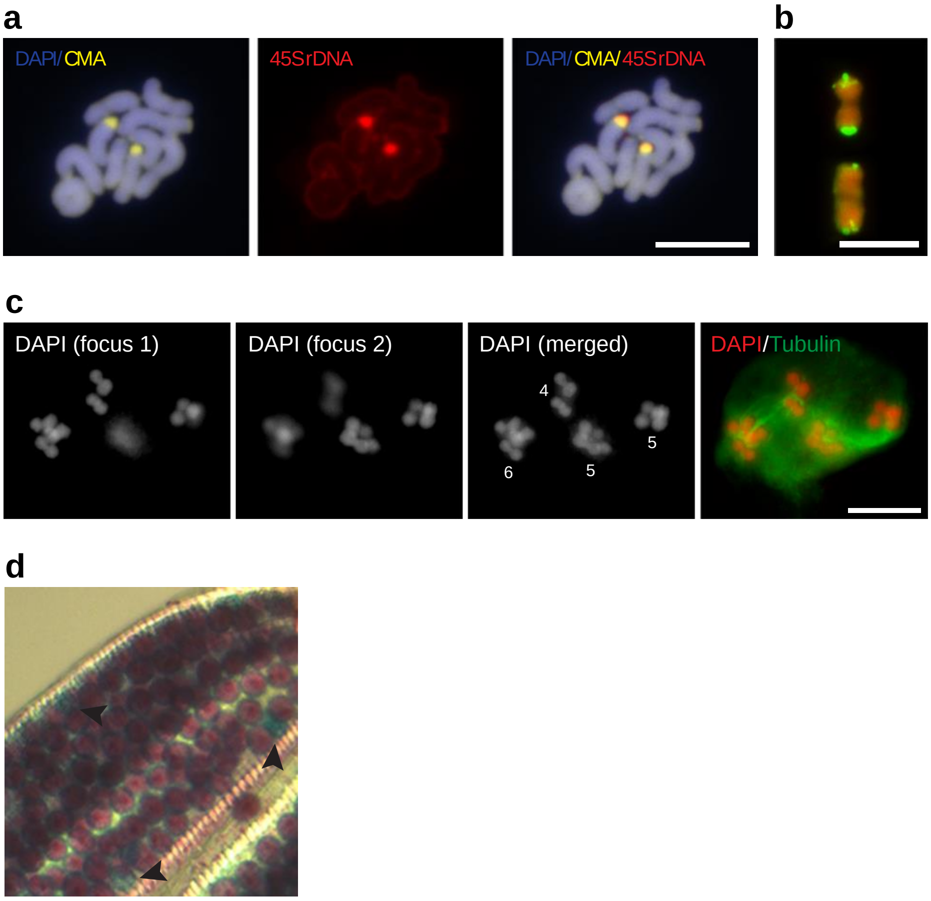

Supplementary Figure 2. 45S rDNA in *R. pubera*. a) Images show a mitotic metaphase cell of *R. pubera*. The CMA signal (yellow; left panel) and the 45S rDNA signal (red; middle panel) co-localize (right panel) at the telomeres of one chromosome pair. **b**) *in situ* hybridisation of 45S rDNA on rod bivalents in meiotic metaphase I to demonstrate the orientation of chromatids within the bivalent. 45S rDNA regions are located at the distal ends of rod bivalents. Chromosomes in red, 45S loci in green. **c**) Mis-segregation in meiosis II. Different focal planes of a late anaphase II stage with two unbalanced products (4 and 6 chromatids) and two balanced products (5 and 5 chromatids). Chromosomes in red, tubulin in green. **d**) Detail of an anther with viable pollen in magenta and non-viable pollen in green (arrowheads). Size bar in (b) corresponds to 5µm and in (a) and (c) to 10µm.

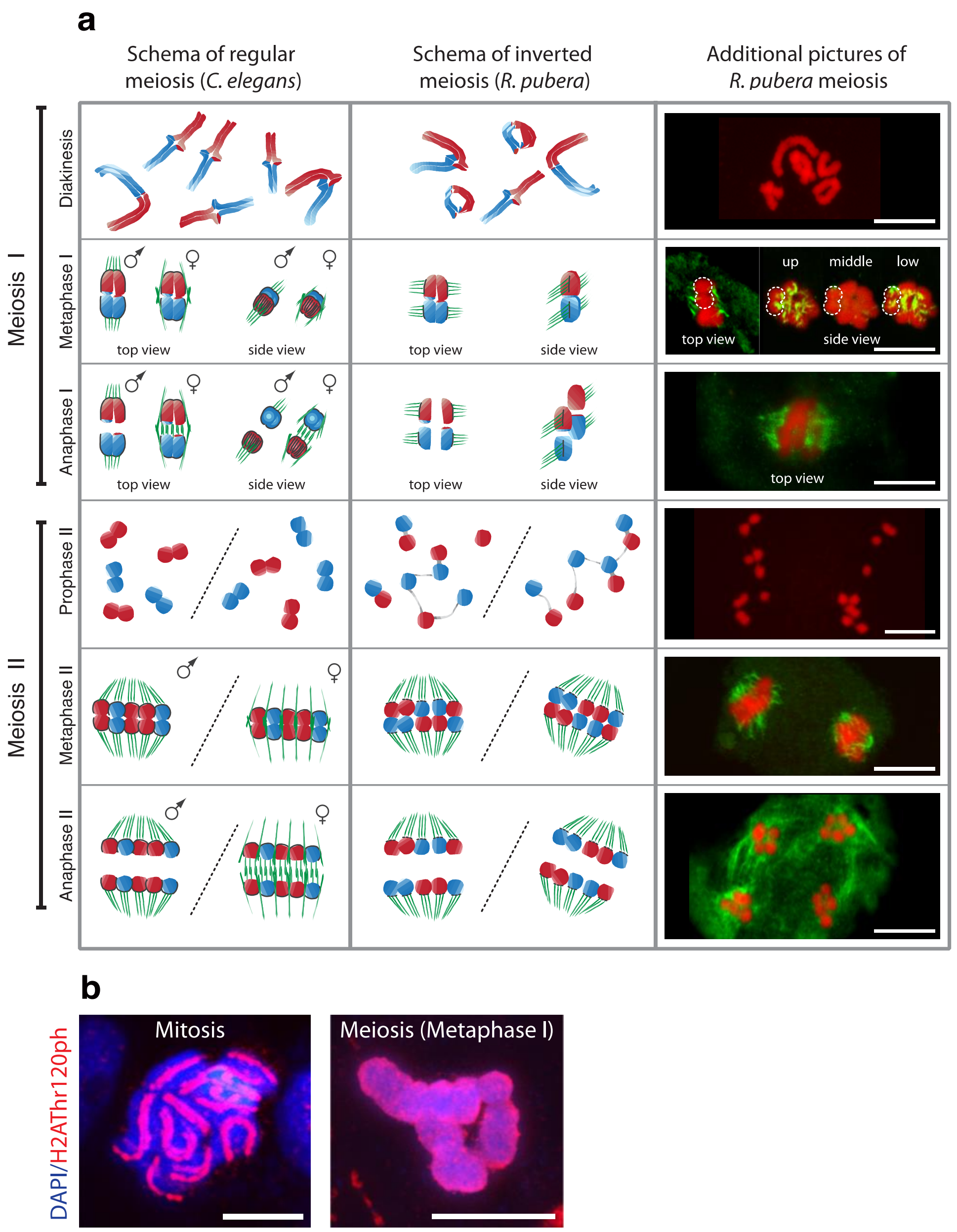

Supplementary Figure 3. Schema of regular and inverted meiosis. a) Illustration of regular meiotic progression in *C. elegans* (left row) and the inverted sequence of events in *R. pubera* (middle row). During meiosis I chromosomes recombine, pair and bivalents with chiasmata become visible in diakinesis. At metaphase I, (only one, rod-shaped bivalent is shown) chromosomes align at the metaphase plate. While in *C. elegans* sister chromatids are mono-oriented towards the same side of the spindle and

the meiotic spindle forms a barrel around the bivalents (female) or shows terminal associations (male), sister chromatids of *R. pubera* show amphitelic attachment to the spindle. At anaphase I, *C. elegans* homologs are separated while in *R. pubera* sister chromatids are separated from each other and pulled to different poles. At prophase II (each half of the dyad shown), sister chromatids of *C. elegans* are still held together by cohesins (not shown). In contrast, the non-sister-chromatids of *R. pubera* appear individualized with some being connected with thin chromatin threads. Importantly, the diploid number of DAPI stained bodies can clearly be visualized in each half of the dyad in prophase II, following the expectations of sister separation during meiosis I. In *C. elegans* at metaphase II/anaphase I, sister chromatids align and are subsequently separated. In *R. pubera*, chromatids associate with the help of an unknown mechanism and subsequently undergo disjunction during anaphase II. The model idealises regular disjunction in meiosis II but actually 19,5% of all meiotic products of *R. pubera* show irregularities. Chromosomes/chromatids are in red and blue, the spindle in green and chromatin threads in grey. Additional images of *R. pubera* meiosis, corresponding to the shown stages (right row), with chromosomes in red and tubulin in green. The size bars correspond to 10µm.

b) H2AThr120ph distribution in *R. pubera*. Mitotic (left panel) and meiotic (right panel) chromosomes (blue) stained with an antibody directed against H2AThr120ph (magenta). While during mitotic metaphase H2AThr120ph appears to be distributed along the chromosomes, defining holocentric kinetochore regions, its distribution during meiotic metaphase I is less specific. Size bar corresponds to 5µm for mitosis and 10µm for meiosis.

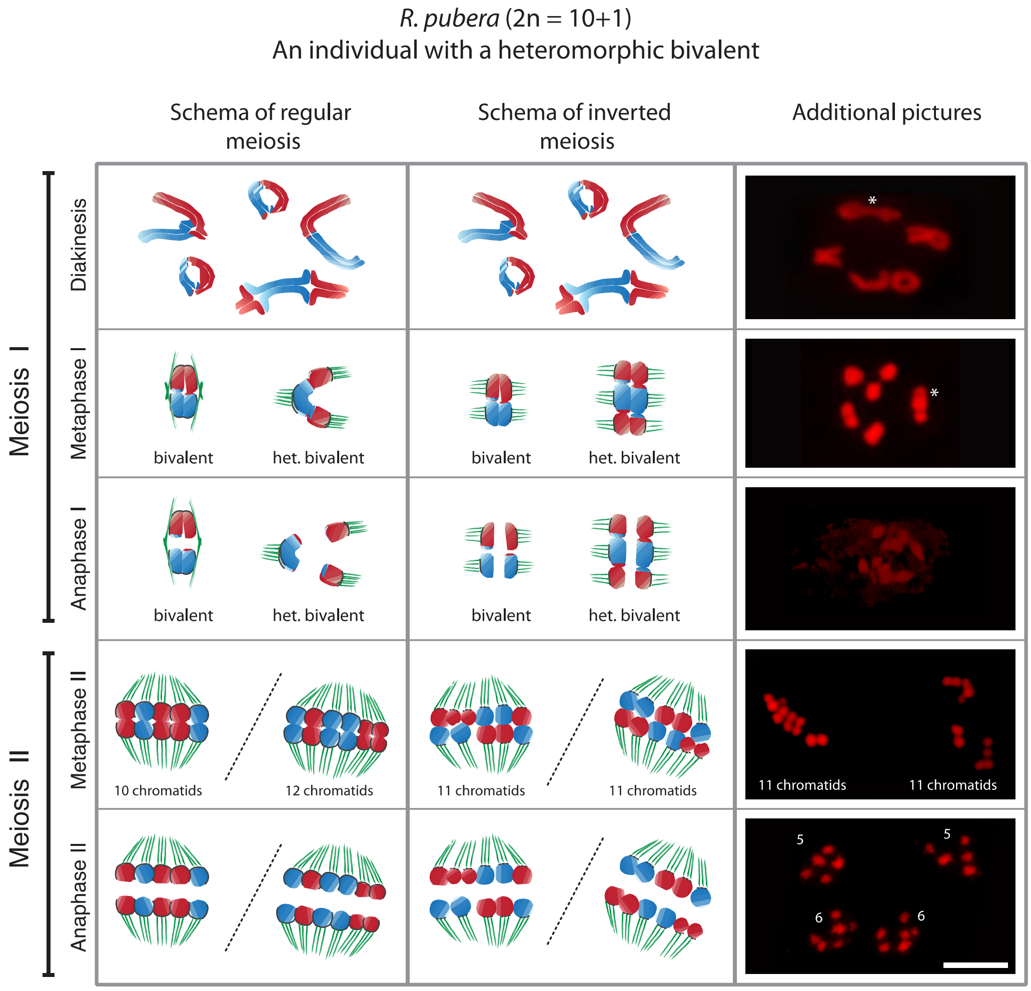

Supplementary Figure 4. *R. pubera* plant with heteromorphic chromosome pair. The figure compares a hypothetical, regular meiotic sequence (left row) with the predictions for an inverted meiotic sequence (middle row) in a *R. pubera* individual with a broken chromosome. During meiosis I chromosomes recombine and pair. At diakinesis, regular bivalents and also one heteromorphic bivalent (het. bivalent) become visible. The latter is comprised of the intact chromosome pairing with the two broken chromosome parts of the homolog (according to our observations, both fragments were always paired with the corresponding intact partner; n = 45). In metaphase I, (only one, rod-shaped, regular bivalent and the trivalent are shown)

chromosomes align at the metaphase plate. In a hypothetical regular meiosis the spindle will either attach to both parts of the broken chromosome (shown) or only to one part of the broken chromosome (not shown) and this will then lead to unequal numbers of chromosomes/fragments in the resulting dyad. In case of an inverted sequence of events in the holocentric plant *R. pubera*, both sisters of the broken chromosome fragments will be distributed to either part of the resulting dyad, yielding balanced numbers of chromatids/fragments. Additional images of *R. pubera* (2n= 10+1) meiosis, corresponding to the stages shown in the cartoon, are provided (right row). Chromosomes are shown in red and the heteromorphic bivalent is highlighted with an asterisk. Importantly, the expectations of inverted meiosis with segregation of sister chromatids at anaphase I (11 chromatids/fragment at each side of the dyad/metaphase II) are always met (n = 24). Refer to text for further details. Parental chromosomes/chromatids are in red and blue and the spindle in green. The size bar corresponds to 10µm.

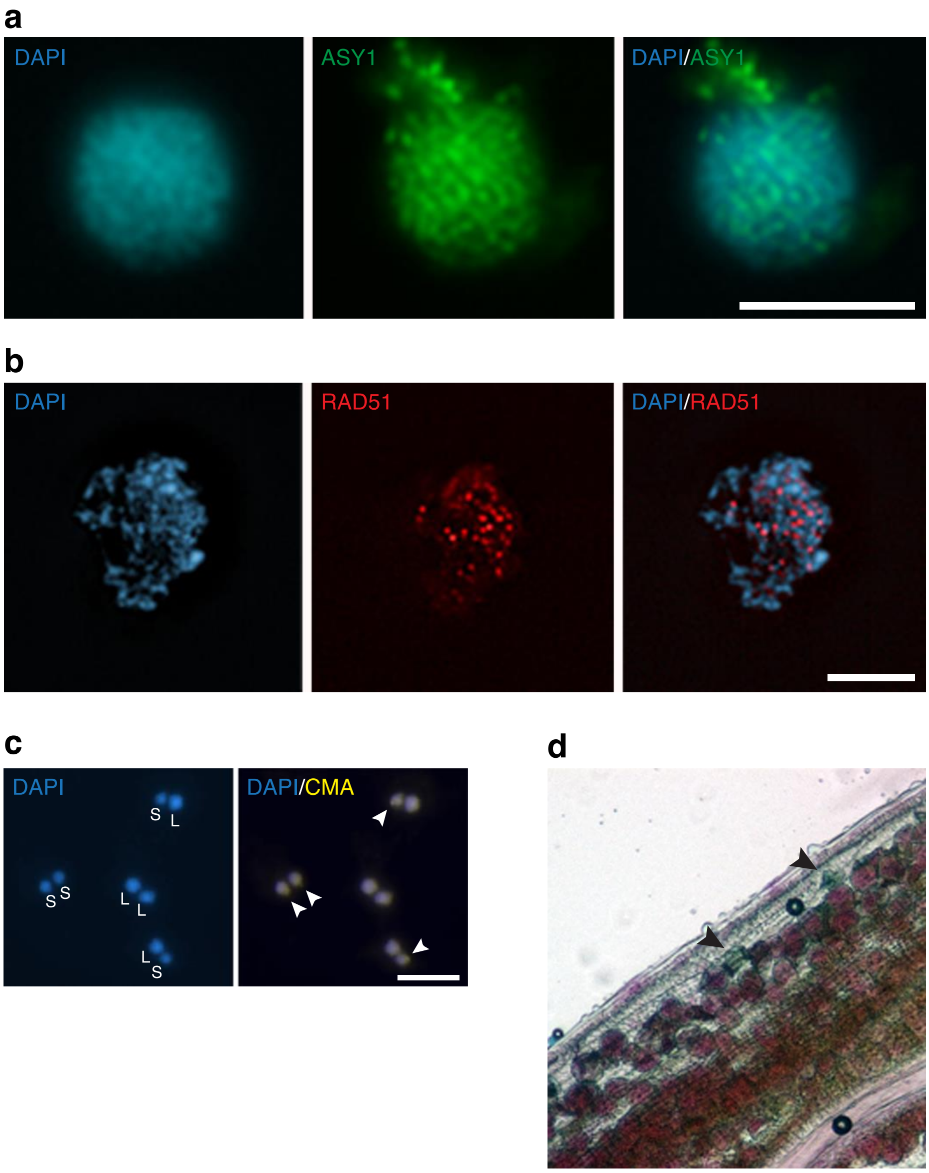

Supplementary Figure 5. Additional data for *Rhynchospora tenuis.* a) Localization of ASY1 (green) in a leptotene nucleus indicates the formation of a meiotic chromosome axis. **b**) Zygotene nucleus with RAD51 (red) foci indicating the occurrence of DNA double strand breaks and subsequent processing. **c**) Mis-segregation in meiosis II. Small (S) and large (L) chromatids are indicated and their identity further corroborated by CMA staining (arrowheads). Two meiosis II products with regular disjunction (SL) and two products with irregular disjunction (SS and LL,

respectively) are shown. **d**) Detail of an anther with viable pollen in magenta and non-viable pollen in green (arrowheads). Size bars in (a) and (b) correspond to 5μm and in (c) to 10μm.

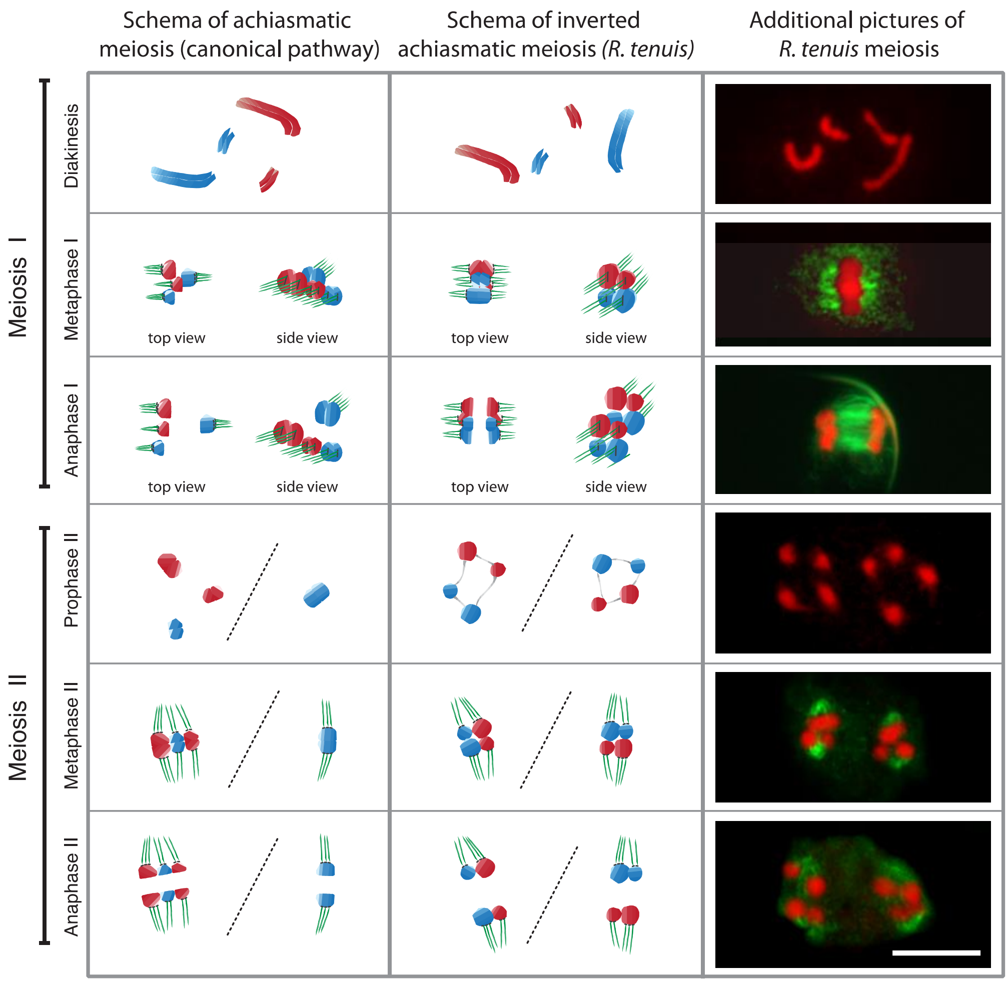

Supplementary Figure 6. Schema of achiasmatic meiotic division patterns. The figure compares a hypothetical achiasmatic meiotic progression embedded in a regular meiotic sequence (left row) with the predictions for an achiasmatic meiosis embedded in an inverted meiotic sequence (middle row). The 4 chromosomes of *R. tenuis* do not form chiasmata and univalents become visible at diakinesis. In the canonical meiotic pathway, univalent are expected to be randomly distributed at anaphase I. In contrast, in case of inverted meiosis sister chromatids become individually attached to the spindle and are separated during anaphase I. The expectation is, to see 4 chromatids in both parts of the resulting dyad and also error-

free, reliable disjunction. Indeed, these expectations are always met (n = 107) (compare with images in the right row at prophase II, and also with data presented in Fig. 5). All four chromatids of *R. tenuis* appear interconnected by thin chromatin threads. In metaphase II, chromatids align and subsequently undergo disjunction during anaphase II. In the case of achiasmatic meiosis, following the canonical pathway, the second meiotic division is expected to resemble an equational, error-free division. In contrast, the second division of an inverted meiosis is expected to face the problem of distributing homologous non-sister chromatids. Depending on the accuracy of a hypothetical mechanism to promote regular disjunction, errors during chromatid disjunction are expected in the second meiotic division of an inverted meiosis. Indeed, about 30% of all meiotic products of *R. tenuis* show irregularities. These results strongly support the claim of inverted meiotic events in *R. tenuis*. Refer to text for further details. Parental chromosomes/chromatids are in red and blue, the spindle in green and chromatin threads in grey. Additional images of *R. tenuis* meiosis, corresponding to the stages shown in the cartoon, are provided (right row), with chromosomes in red and tubulin in green. The size bar corresponds to 10µm.
